# Applications of Explainable Artificial Intelligence in Diagnosis and Surgery

**DOI:** 10.3390/diagnostics12020237

**Published:** 2022-01-19

**Authors:** Yiming Zhang, Ying Weng, Jonathan Lund

**Affiliations:** 1 School of Computer Science, Faculty of Science and Engineering, University of Nottingham Ningbo China, Ningbo 315100, China; yiming.zhang2@nottingham.edu.cn; 2 School of Medicine, University of Nottingham, Nottingham NG7 2RD, UK; jon.lund@nottingham.ac.uk

**Keywords:** artificial intelligence, machine learning, deep learning, explainable artificial intelligence (XAI), diagnosis, surgery

## Abstract

In recent years, artificial intelligence (AI) has shown great promise in medicine. However, explainability issues make AI applications in clinical usages difficult. Some research has been conducted into explainable artificial intelligence (XAI) to overcome the limitation of the black-box nature of AI methods. Compared with AI techniques such as deep learning, XAI can provide both decision-making and explanations of the model. In this review, we conducted a survey of the recent trends in medical diagnosis and surgical applications using XAI. We have searched articles published between 2019 and 2021 from PubMed, IEEE Xplore, Association for Computing Machinery, and Google Scholar. We included articles which met the selection criteria in the review and then extracted and analyzed relevant information from the studies. Additionally, we provide an experimental showcase on breast cancer diagnosis, and illustrate how XAI can be applied in medical XAI applications. Finally, we summarize the XAI methods utilized in the medical XAI applications, the challenges that the researchers have met, and discuss the future research directions. The survey result indicates that medical XAI is a promising research direction, and this study aims to serve as a reference to medical experts and AI scientists when designing medical XAI applications.

## 1. Introduction

Machine learning (ML) and deep learning (DL) have achieved impressive progress recently, and the success of artificial intelligence (AI) in the medical field has resulted in a significant increase in medical AI applications. The goal of medical AI research is to build applications that use AI technologies to assist doctors in making medical decisions [1]. AI is used in various medical applications, such as disease diagnosis [2], surgery [3], and many more. However, medical AI applications are faced with some challenges, including the black-box nature of some AI models. The poor explainability of these black-box models leads to distrust from medical experts to make explainable clinical inferences. There are often millions of parameters in DL models, and they only return a final decision result without any explanation. Due to the lack of transparency of deep neural networks, it is hard for the user to judge whether the decision is reliable, compromising trust with doctors. Medical AI applications need to be transparent to increase the level of trust with doctors. Research on explainable artificial intelligence (XAI) has recently gained considerable attention [4]. For medical AI applications to be accepted and integrated into practice, XAI is crucial.

### 1.1. Related Artificial Intelligence Concepts

Artificial intelligence refers to the development of intelligence by machines, and machine learning is a part of AI. An ML algorithm is trained by the provision of many examples for a given task, the statistical pattern in these examples is found, and eventually, the rules to automate the task are discovered [5]. Traditional ML algorithms, including k-nearest neighbor (kNN), support vector machine (SVM), decision tree (DT), and random forest (RF), have been applied in medical AI. kNN is an algorithm that finds the closest data points in the training set to be the prediction for the new data [6]. SVM assumes that the data are linearly separable and seeks to find a linear hyperplane (decision boundary) to separate the data. The examples in an SVM model are represented as points in space, divided into separate categories by a linear hyperplane [7]. A decision tree is a tree-like structure in which each internal node represents an attribute test, each branch represents an outcome of the test, and each leaf indicates the class. In DT, the basic idea is to break up a complex decision into several simpler ones so that the final result will resemble the intended desired outcome [8]. RF is an ensemble machine learning algorithm that consists of many decision trees. For classification tasks, the decision of RF is the voting result from these decision trees [9].

In addition to traditional machine learning methods, many studies have also used deep learning methods for medical applications. A deep learning algorithm can learn representations of raw data without feature engineering. Typical deep learning methods include multi-layer perceptron (MLP), deep neural networks (DNNs), convolutional neural networks (CNNs), and recurrent neural networks (RNNs) [10]. Additionally, in terms of the performance evaluation of the ML methods, the typical evaluation metrics are accuracy, precision, recall, F1-score, AUC, and ROC [11].

### 1.2. Related Explainable Artificial Intelligence Concepts

**Figure 1:**
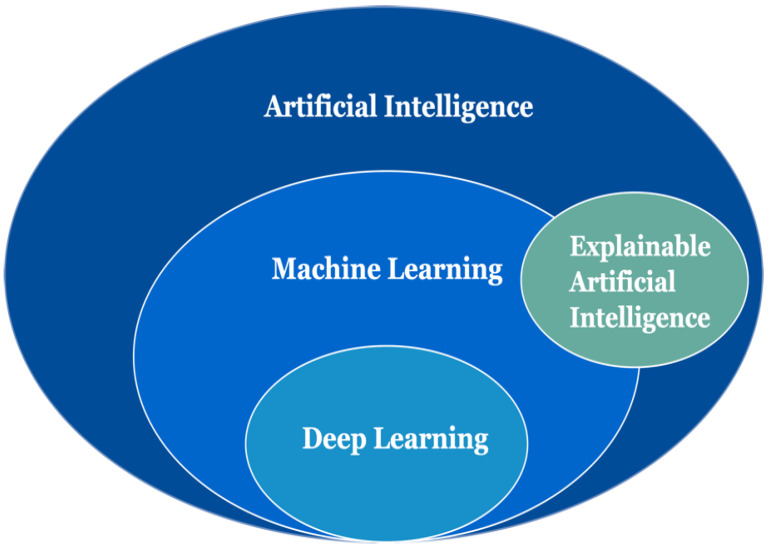
The relationship between artificial intelligence, machine learning, deep learning, and explainable artificial intelligence.

According to [12], explainability is the ability to explain AI decision-making in understandable terms for humans, with a broader range of end-users on how a decision has been drawn. The different end-users focus on the different perspectives of explainability. AI experts or data scientists are more concerned about the explainability of the model/algorithm. However, medical experts or physicians are more concerned about clinical inference/prediction. The other notion related to explainability is interpretability. Interpretability means the capacity to provide the meaning of an abstract concept [13]. Explainability refers to the interpretation of predictions made in the presence of new cases, whereas interpretability refers to the rendition of the model learned from the data during training [14]. Furthermore, there are two types of XAI methods: intrinsic and post hoc [15]. An intrinsic method is one in which we can understand a decision-making process or the basis of a technique without additional information. Typical intrinsic methods include linear regression (LR) [16], logistic regression, k-nearest neighbor, rule-based learners, general additive models, Bayesian models, and decision trees. Deep learning is a subset of machine learning, and machine learning is a subset of AI. In addition, we believe that XAI is a subset of AI, and its intrinsic methods are ML. Hence, in Figure 1, we show the relationship between artificial intelligence, machine learning, deep learning, and explainable artificial intelligence.

**Figure 2:**
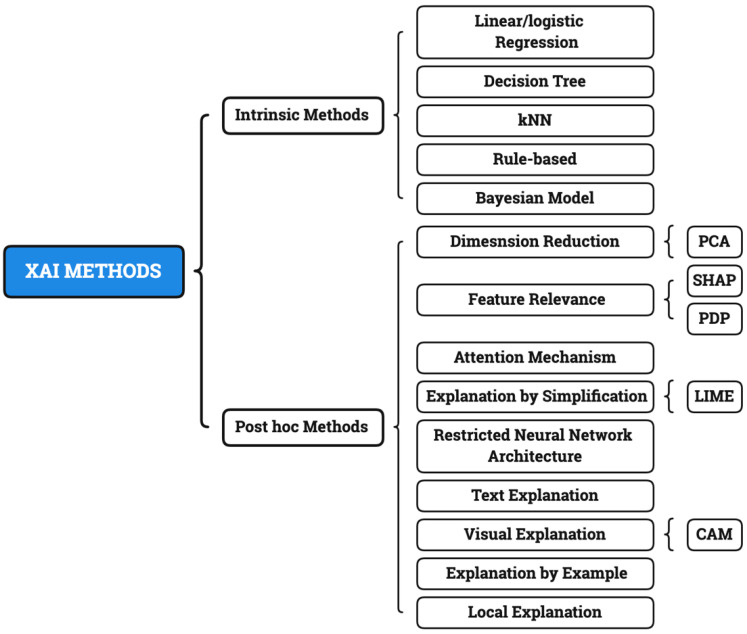
Taxonomy of XAI methods, post hoc XAI types, and some examples.

Using this method, we can understand what part of the input data accounts for the classification decision in any classifier. Other post hoc methods include SHapley Additive exPlanations (SHAP) [17], class activation mapping (CAM) [18], principle component analysis (PCA) [19] and Gradient-weighted class activation mapping (Grad-CAM) [20]. According to [21,22], post hoc explainability methods can be categorized as: dimension reduction, attention mechanism, restricted neural network architecture, text explanation, visual explanation, local explanation, explanation by example, explanation by simplification and feature relevance. The taxonomy of XAI methods is shown in Figure 2.

For evaluating XAI, no objective or unified evaluation metric has been adopted. Doshi-Velez and Kim define three types of XAI evaluation approaches [12]: application-grounded evaluations, human-grounded evaluations, and functionally grounded evaluations.

### 1.3. Contributions

**Figure 3:**
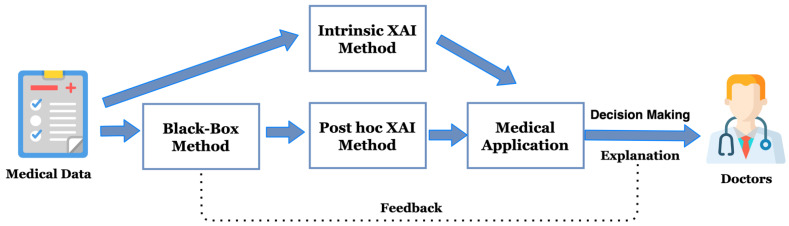
The overall pipeline of a medical XAI application: the XAI methods can be intrinsic or post hoc, and they can provide decision-making and explanation to the doctors.

There have been few studies exploring XAI’s potential in medical AI applications [23]. This study is focused on the medical XAI applications in diagnosis and surgery. The general pipeline of a medical XAI application is shown in Figure 3. As shown in Figure 3, using the intrinsic XAI method enables the medical XAI application to examine the medical data and provide decisions and explanations to the doctors. Alternatively, if the medical application utilized post hoc XAI, the black-box methods would be applied to medical data for decision-making, followed by the post hoc XAI providing an explanation of the black-box methods.

In recent years, the importance of XAI has been widely recognized in academia and industry. Due to the high degree of complexity, the decision-making process of the deep learning model is hard to interpret. Moreover, the black-box nature of these models is dangerous if they are deployed in clinical applications because they may not provide reliable justification to the medical experts. Many XAI studies have been proposed in the AI community to overcome this issue. However, in the interdisciplinary field of artificial intelligence and medicine, deep learning models are the majority choice for most medical AI applications. Therefore, it is essential to utilize and develop XAI methods instead of deep learning black-box methods. We found that most surveys on medical AI applications only use deep learning, but there has been no survey focusing on the medical AI applications using XAI, especially in diagnosis and surgery. We believe that such a survey will give both medical and AI experts insights into the recent progress on medical XAI applications. Furthermore, it will be helpful for medical and AI researchers to develop their medical XAI applications. This survey aims to address the following three research questions (RQs): (RQ1) What are the current research trends on medical XAI applications?; (RQ2) How do the studies included in this survey tackle the trade-off between accuracy and explainability?; and (RQ3) Is it possible to deploy these models into the clinical real-world environment to assist the medical experts and make an explainable clinical inference? In summary, the main contributions of this survey include:

- A brief introduction of the AI/DL concepts, XAI concepts, and the general pipeline of medical XAI applications gives a quick start for medical experts;
- Our survey also provides a recent three-year overall literature review on the medical XAI applications in the fields of diagnosis and surgery with a thorough analysis;
- We summarize the current trends, as well as discuss the challenges and the future directions on how to design a better medical XAI application.

The rest of the paper is structured as follows: Section 2 describes the survey’s search strategy; Section 3 presents the study selection results of the medical XAI applications on diagnosis and surgery; in Section 4, we present the discussion of the survey, including the findings of the study, experimental showcase, challenges, limitations, research gaps, as well as the future directions and answers of the research questions; Section 5 concludes the survey.

## 2. Search Strategy

A literature search was conducted using keywords explainable artificial intelligence, diagnostics, and surgery. Additionally, the research papers cited in this review were found on three electrotonic databases—PubMed, IEEE Xplore, and Association for Computing Machinery (ACM)—for relevant publications between 2019 and 2021 inclusive. Google Scholar is searched between these dates for potentially relevant studies as well. The search strings we use in this survey: (ALL(“Explainable Artificial Intelligence”) OR ALL(“XAI”) OR ALL(“Explainable AI”) OR ALL(“Diagnostics”) OR ALL(“Surgery”) OR ALL(“Medical”)). The survey aims to identify publications on medical XAI applications in diagnosis and surgery. Hence, all the included papers had to focus on this topic. We excluded all the survey/review articles, non-English articles, or non-peer-reviewed articles. The titles and abstracts of these research papers were then screened for eligibility. Next, all screened research papers were reviewed for relevance in full text; the eligible papers are included in this review.

## 3. Medical Explainable Artificial Intelligence Applications

### 3.1. Diagnosis

In [24], a computer-aided framework was proposed by Kavya et al. for allergy diagnoses. The authors applied several ML algorithms and then selected the best-performing algorithm using k-fold cross validation. In terms of the XAI method, they developed a rule-based approach by building a random forest. More specifically, each path in a tree is represented as an IF-THEN rule, and the explanations are extracted from medical data. Additionally, the authors deployed the computer-aided framework on the mobile application, which can assist junior clinicians in confirming the diagnostic predictions. In [25], Amoroso et al. presented an XAI framework for breast cancer therapies. They applied the clustering and dimension reduction method, and the experiment results demonstrated that the framework could outline the most important clinical feature for the patient and designed oncological therapies. Dindorf et al. proposed an explainable pathology-independent classifier for spinal posture [26]. The authors used SVM and RF as the ML classifiers and then applied LIME to explain the prediction of the ML classifier. In [27], EI-Sappagh et al. proposed an RF model for Alzheimer’s disease (AD) diagnosis and progression detection. In addition, the authors first applied SHAP to select the critical features in the classifier. Then, the authors used a fuzzy rule-based system. SHAP could provide a local explanation for specific patient diagnosis/progression prediction explanations about feature impacts. In addition, the fuzzy rule-based system could generate natural language forms to help patients and physicians to understand the AI model. In [28], Peng et al. proposed an XAI framework that can assist doctors with the prognosis of hepatitis patients. In this paper, the authors compared intrinsic XAI methods such as logistic regression (LR), decision tree (DT), and kNN with the complex models SVM, XGBoost, and RF. Furthermore, the authors applied the post hoc methods SHAP, LIME, and partial dependence plots (PDPs) [29].

**Figure 4:**
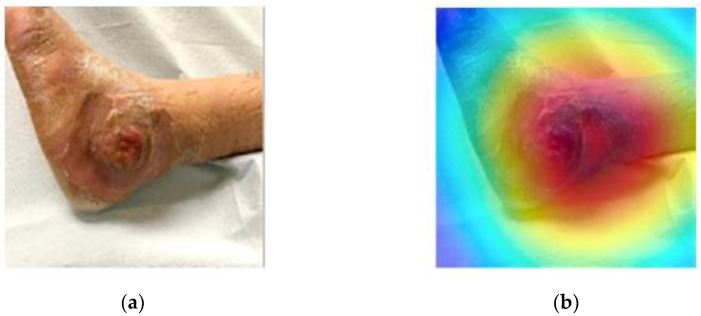
Chronic wound image and its importance map using LIME [30]: (**a**) original wound image; (**b**) importance map.

In [30], Sarp et al. first proposed a CNN-based model for chronic wound classification and then applied the XAI method LIME to explain the CNN-based model. The proposed CNN-based model also used the transfer learning technique and achieved an average precision at 95%, an average recall at 94%, and an average F1-score at 94%. The original wound image and its heatmap image produced by LIME are shown in Figure 4. By using LIME, the model could provide visual cues for clinician. Tan et al. presented an otosclerosis-logical neural network (LNN) on temporal high-resolution computed tomography (HRCT) bone slices for fenestral otosclerosis diagnosis [31]. The proposed method achieved an AUC of 99.5% on the external test dataset. Additionally, they applied the XAI method to visualize the learned deep representations of the LNN model. In [32], Wu et al. proposed a counterfactual multi-granularity graph supporting fact extraction (CMGE) for lymphedema diagnosis. CMGE is a graph-based neural network that can extract facts from the electronic medical record (EMR). In addition, it could obtain a causal relationship between features. The proposed model was evaluated on actual Chinese Electronic Medical Records, and demonstrated an accurate and interpretable approach by providing counterfactual reasoning on the graph.

Similarly, Chen et al. presented an interpretable clinical diagnosis model on the EMR documents [33]. Additionally, the proposed model consisted of Bayesian network ensembles and entity-aware CNN networks with an accuracy of Top-3 prediction of over 88%. More specifically, the explainability of the Bayesian network in the model was achieved by building connections between diseases and symptoms. Then, three certificated physicians evaluated the explanation of the model by reviewing the extracted relationships in the medical knowledge graph. In [34], Rucco et al. combined the topological and textural features and presented an XAI application to diagnose glioblastoma. In addition, the authors validated the proposed AI model on the fluid-attenuated inversion recovery (FLAIR) for glioblastoma multiforme (GBM) classification. In terms of explainability, the authors used LIME XAI methods to compute the local feature relevance to samples in the test set. In [35], Gu et al. proposed a computer-aided diagnosis system named VINet to provide diagnostic visual interpretations. The proposed VINet achieved an 82.15% classification accuracy on a computed tomography image dataset (LUNA16). Furthermore, the authors compared VINet with other XAI methods such as CAM, visual back-propagation (VBP), and layer-wise relevance propagation (LRP). Moreover, it could demonstrate SOTA visual interpretations.

In [36], Kroll et al. developed a grammatical evolution-based framework for Alzheimer’s disease (AD) diagnosis and prognosis. The proposed framework was evaluated on a magnetic resonance imaging (MRI) dataset, and the experimental results showed that it could provide both accuracy and explainability. The grammatical evolution could generate patterns of strings according to the production rules. In terms of explainability, the authors utilized grammatical evolution for feature representation and then applied them in classification. Meldo et al. proposed a lung cancer computer-aided diagnosis system with explanation sentences [37]. The proposed system consists of two parts: the first part is a local post hoc XAI model using LIME; the second part transforms the selected the important features into natural language. In [38], Yeboah et al. presented an ensemble clustering-based XAI model for traumatic brain injury (TBI) prognostic and diagnostic analysis. In addition, the proposed explainable framework can combine automated data analytics and medical expert knowledge. Regarding the interpretation, the framework utilized quality assessment of the clustering features, the discriminant features identification and clinical interpretation. In [39], Wang et al. proposed a CNN-based model named COVID-Net for COVID-19 case detection using chest X-ray (CXR) images. The authors also compared COVID-Net with VGG-19 and ResNet-50. COVID-Net achieved a 93.3% accuracy on the COVIDx test dataset and 91% sensitivity. Additionally, the authors applied the XAI method GSInquire [40] to investigate the prediction of the COVID-Net. GSInquire could be used to obtain improved insights into neural networks and can also learn to generate networks.

In [41], Sabol et al. proposed an XAI application named the cumulative fuzzy class membership criterion (CFCMC) that could assist pathologists and be used for colorectal cancer diagnosis. The proposed system provides explainability through a semantical explanation of the tissue type classification results. In addition, CFCMC showed the original whole slide images (WSI) of the tissue and its corresponding label map visualization. The proposed XAI model was evaluated by 14 pathologists. Wei et al. designed an AI-assisted diagnosis of thyroid nodules and then tested the model for classification performance [42]. Additionally, the authors applied data pre-processing techniques to localize and diagnose thyroid nodules. Through conducting experiments, they found that the A/T ratio and margin information of thyroid nodules are important clinical features for thyroid nodules diagnostics. The authors utilized class activation mapping to visualize the proposed CNN-based neural network for model explainability. CAM uses global average pooling and a fully connected layer to visualize the neural network and display the most important features. In [43], Chang et al. presented an explainable deep neural network (EDNN). The model was trained on a dataset with 200 schizophrenic patients and healthy controls in the Taiwan Aging and Mental Illness (TAMI) cohort. Using the TAMI cohort, the proposed framework achieved an 84.0% accuracy in gray matter (GM) and 90.22% accuracy in white matter (WM). In terms of explainability, the system provided a three-dimensional (3D) visualization of the subject’s brain imaging data that could optimize the diagnostic process. In [44], Magesh et al. proposed a CNN-based model for early Parkinson’s disease (PD) detection. The dataset in the paper consisted of 642 single-photon emission computed tomography (SPECT) images from the Parkinson’s Progression Markers Initiative (PPMI) database. Furthermore, the authors utilized transfer learning on the CNN-based model. The post hoc XAI method LIME was used for interpretation in the research. LIME could emphasize the regions of interest in the SPECT image with the impact areas that classify the data as healthy controls.

In [45], Cho et al. proposed an interpretable machine learning method to predict post-stroke hospital discharge disposition. The authors selected linear model logistic regression (LR) as the baseline model and then compared it with the black-box model, including RF, RF with AdaBoost, and MLP. To interpret the black-box model, the author utilized LIME and provided explanations for the prediction. By using LIME, the authors identified the most important features for the model. More specifically, features such as age, diabetes, and source of admission were important for post-stroke hospital discharge disposition prediction. Lamy et al. presented a visual case-based reasoning (CBR) approach for breast cancer diagnosis [46]. CBR used a database of previously solved cases to determine the answer to a query case. This is a form of analogical reasoning. From the database, similar cases were retrieved, and their solutions were then adapted to the query. The proposed automatic rainbow boxes-inspired algorithm (RIBA) was compared with kNN and distance-weighted kNN (WkNN). Additionally, the proposed method achieved an 80.3% accuracy on a real breast cancer dataset. Moreover, medical experts evaluated the proposed XAI application and they found the visual CBR approach to be very appealing. In [47], Das et al. proposed an XAI model for Alzheimer’s disease (AD) diagnosis, named a sparse high-order interaction model with rejection option (SHIMR). The proposed SHIMR was validated on the AD dataset, and shown to have high accuracy, interpretability, and cost-effectiveness. Using SHIMR, highly accurate and interpretable decision sets could be created, with collections of “if–then” rules that reflected the higher-order interactions between a set of individual features could be discovered.

### 3.2. Surgery

**Figure 5:**
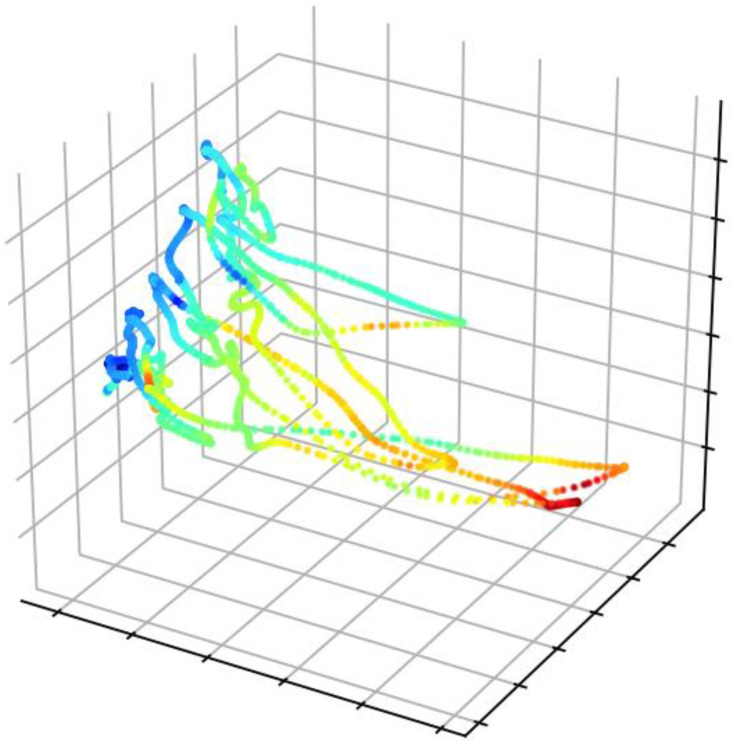
Visual feedback of the surgeon’s surgical task using CAM [50]. Visual feedback for the surgeon’s surgical task using CAM [50]. The red and orange subsequences in the plot show the high contribution to the surgeon’s surgical skill assessment task. In contrast, the green and blue subsequences indicate the low contribution.

In [48], Yoo et al. presented a multiclass XGBoost model to select the laser surgery option at an expert level. The authors validated the proposed method on the subjects who had refractive surgery at the B&VIIT Eye Center and achieved a 78.9% accuracy on the external validation dataset. This also provides a clinical understanding of the machine learning method using the SHAP technique. In [49], Mirchi et al. proposed a framework that can be used for surgical training with automated educational visual feedback. The authors trained and evaluated an SVM model on the simulated medical and surgical data and achieved an accuracy of 92%, a specificity of 82%, and a sensitivity of 100%. Additionally, they provided a thorough explanation of the proposed machine learning algorithm by identifying the teachable metrics that contribute to the classification. Fawaz et al. presented an accurate and interpretable surgical skill assessment medical application by training a fully convolutional neural (FCN) network to classify surgical skill levels using surgical kinematics [50]. The proposed model achieved state-of-the-art performance on the JIGSAWS public dataset for three surgical skill tasks. Moreover, the authors applied the CAM to provide interpretable classification feedback. The visual feedback provided by the CAM is shown in Figure 5 below. CAM is a visual explanation post hoc XAI method used for CNN-based models to locate the features in the CNN that influence classification decisions. More specifically, CAM uses a global average pooling (GAP) layer after the convolutional layer with the possibility to visualize which trial parts contribute most to skill classification. Through investigation of the behavior specific to a skill level, observers could identify motion patterns characteristic of a particular class of surgeons.

In [51], Kletz et al. proposed a CNN-based medical application that can learn the representation of instruments in laparoscopy, and they validated the model on various datasets. They also provided activation maps of different CNN layers to help understand how the model classified the instrument. An explainable AI system, XAI-CBIR, was proposed by Chittajallu et al. for surgical training [52]. XAI-CBIR is an explanation by example post hoc XAI method. It provides explanations by extracting the representative examples. More specifically, it exploits a self-supervised deep learning model to extract semantic descriptors from MIS video frames. In addition, it used a saliency map to provide a visual explanation as to why the system believes that the retrieved image is similar to the query image. By utilizing the XAI-CBIR system, minimally invasive surgery (MIS) videos can be retrieved based on their content. The proposed system was evaluated on the Cholec80 dataset, and the percentage of relevant frames among the top 50 retrieved frames for three phases were 64.42%, 99.54%, and 99.09%, respectively. In addition, they applied a saliency map to guide relevance feedback with visual explanations.

## 4. Discussion

### 4.1. Current Research Trends

Artificial intelligence techniques such as deep learning have recently played a revolutionary role in healthcare, including diagnosis and surgery. These techniques have been shown to be effective in these fields. The accuracy of some deep-learning-based diagnosis tasks even surpasses human medical experts. However, the black-box nature of the deep learning model limits the explainability of these models and limits their practical deployment in medicine. In the interdisciplinary field of artificial intelligence and medicine, many researchers have realized that the key of AI deployment in the clinical environment is not the accuracy of the model, but the explainability of the AI model. Medical AI applications should be explained before being accepted and integrated into the medical practice. Hence, the acceptance of medical AI applications requires explainable artificial intelligence, and there is motivation to survey the medical XAI.

In this survey, we included 27 papers in diagnosis and surgery using explainable artificial intelligence. As observed in Table 1 and Table 2, the studies included in this survey were analyzed from the perspectives of AI algorithm, XAI method, and AI performance. We found that the most popular traditional machine learning algorithm is random forest, with 25.9% (7/27) of the published papers reported in this survey having conducted experiments with random forest; the most popular deep learning method is convolutional neural networks (CNNs), with 37.0% (10/27) papers utilizing CNN-based model such as VGG-16; furthermore, LIME is the most commonly used XAI approach in these papers, with 25.9% (7/27) of the papers utilizing LIME to explain the proposed machine learning model. For the XAI methods, most of the papers used post hoc methods, and followed the pipeline introduced in Figure 3. They firstly applied deep learning algorithms such as CNN-based models or complex machine learning random forest models and then used post hoc methods such as LIME, SHAP and PDP to explain the AI model; secondly, they built the medical applications and provided decision-making to the doctors. In addition, regrading XAI evaluation, only 11.1% (3/27) of studies applied XAI evaluation.

The summary of this survey suggests that different machine learning methods or deep learning methods would be optimal solutions for various medical XAI applications. There is no unified machine learning model or XAI approach that suit all the diagnosis and surgery task, and it would depend on the dataset size, data type, and many other factors.

### 4.2. Experimental Showcase: Breast Cancer Diagnosis

We have analyzed the current research trends by summarizing the literature included in the survey. In addition, to better understand the XAI methods, we have demonstrated one medical XAI application experimental showcase, which is a breast cancer diagnosis.

#### 4.2.1. Dataset

One of the most common forms of cancer among women is breast cancer. In this paper, we use a real-world breast cancer Wisconsin (Diagnostic) dataset which contains 569 patients [52]. The dataset is publicly available from UCI machine learning repository: https://archive.ics.uci.edu/ml/datasets/Breast+Cancer+Wisconsin+%28Diagnostic%29 (Accessed on 22 December 2021). The dataset features are numerical and extracted from the digitized image of a breast mass’s fine needle aspirate (FNA). In terms of the class distribution, there are 357 benign and 212 malignant.

#### 4.2.2. Experiment Setup

The experiments were performed on a laptop with a 2.6 GHz 6-Core CPU and implemented in Python: 70% of the breast cancer dataset was the training dataset, and 30% of the dataset was the testing dataset. The proposed black-box model was trained using the Scikit-learn toolkit [53]. For XAI methods, we utilize the Python library InterpretML [54].

#### 4.2.3. Intrinsic XAI Method: Rule-Based

Rule-based methods are logical expressions of the form IF-THEN. We implemented a rule based on the breast cancer classification task in this showcase. In addition, we evaluated the model’s performance in terms of accuracy, precision, recall, and F1-score. The proposed rule-based model achieved an accuracy of 60.81%, precision 60.95%, recall 99.04%, and F1-score 75.46%.

#### 4.2.4. Post hoc XAI Method: SHAP

**Figure 6:**
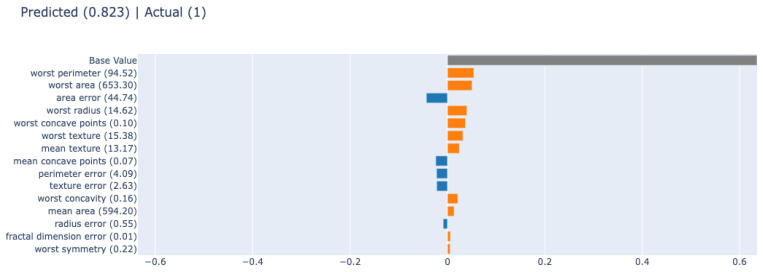
Interpreting a prediction with the post hoc XAI method: SHAP.

The type of dataset was numerical, and we selected a random forest with 300 trees as the black-box model. The random forest achieved an accuracy of 95.91%, precision of 97.09%, recall of 96.15%, and F1-score of 96.62%. The random forest performed better than the rule-based method. In addition, we applied the post hoc method SHAP to explain the black-box model. SHAP is a feature relevance post hoc XAI method inspired by game theory. It aims to increase interpretability by calculating the importance value of each feature for each prediction using Shapley values. As shown in Figure 6, it demonstrates how SHAP interprets the black-box model’s prediction.

#### 4.2.5. Post hoc XAI Method: LIME

**Figure 7:**
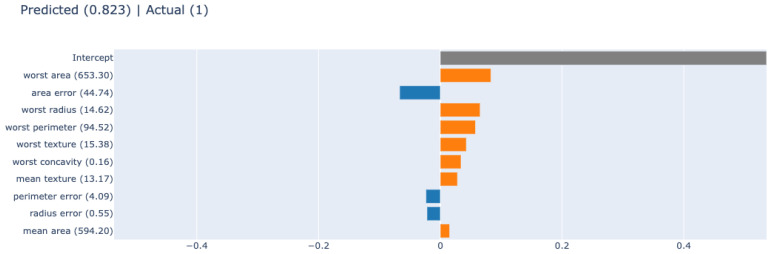
Interpreting a prediction with the post hoc XAI method: LIME. The x-axis shows the feature effect.

LIME is an explanation by simplification post hoc XAI method, which can explain a single prediction generated by any black-box model. It explains a prediction by replacing the complex model with a locally interpretable surrogate model. By focusing on a sufficiently narrow decision surface, LIME attempts to explicitly model the local neighborhood of any prediction. Figure 7 is an example of LIME to interpret a black-box model’s prediction. More specifically, the features “worst area”, “worst radius”, “worst perimeter”, “worst texture”, “worst concavity”, “mean texture” and “mean area” has a positive effect on the prediction.

#### 4.2.6. Post hoc XAI Method: PDP

**Figure 8:**
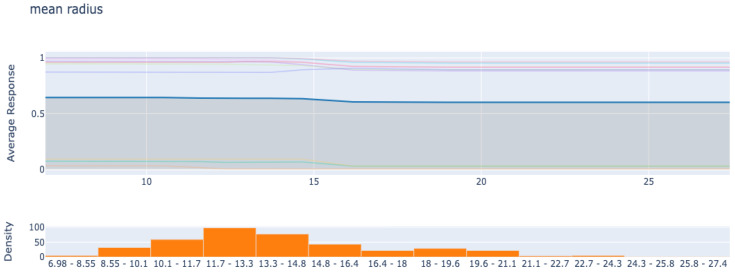
Interpret the black-box model’s decisions with PDP for the feature “mean radius”.

PDP is also a feature relevance explanation post hoc XAI method, which interprets the black-box model by plotting the impact of subsets of features on the model’s predictions. Figure 8 is a PDP visualization of the dependence between the feature “mean radius” and the response.

### 4.3. Challenges, Limitations and Research Gaps

The survey also identified some challenges and limitations. Firstly, in some papers, accuracy was the only machine learning evaluation metric used to evaluate the model performance, which is unreasonable. Using one evaluation metric cannot provide an objective evaluation of the machine learning algorithm. Secondly, the size of these datasets in medical XAI applications was relatively small, and the data quality of these datasets was not guaranteed. Hence, the AI model’s performance may be limited by the small dataset size and low input data quality. The AI model was only trained and validated on the small dataset; therefore, the model was likely to cause an overfitting issue. The generalization of the model was low. Thirdly, in terms of the XAI evaluation, there are still no unified XAI evaluation methods accepted by most researchers in the community. XAI models can only be evaluated qualitatively because evaluation still relies on human cognition. However, most of the papers in this survey only provided the XAI methods without any XAI evaluation. Only a few researchers provided XAI evaluations by medical professionals. Finally, many of the studies only applied the existing machine learning or XAI methods. These AI approaches were designed without any medical experts’ participation, which resulted in these medical XAI applications having a lack of innovation and prior knowledge from the doctors, and they may not meet the doctor’s actual clinical needs.

We also concluded with two research gaps we have found after reviewing the medical XAI literature. Firstly, the majority of studies in the interdisciplinary field of artificial intelligence and medicine focus on deep learning methods, including MLP, CNNs, RNNs, and transformers. These deep learning-based models, such as transformers, contain millions of parameters and are hard to interpret. However, data scientists and AI experts in the interdisciplinary field of artificial intelligence and medicine should focus on XAI rather than SOTA deep learning models. Secondly, medical XAI applications should be evaluated by medical experts. However, most of the medical XAI applications lack XAI evaluation and medical experts’ evaluation. The medical XAI applications should have well-designed HCI and provide a reasonable explanation to medical experts.

### 4.4. Future Directions

For the future research directions, we believe that AI will be applied in many different diagnoses and surgery-related tasks. XAI will play an essential role because it can increase the transparency of these models and gain trust from doctors. To address the above challenges, initially, we think that it would be desirable to consider different evaluation metrics such as specificity and sensitivity accordingly for machine learning evaluation, but not only consider accuracy. Moreover, it would be preferable to use cross-validation to validate the trained model. Next, in the future, we should collect and build the dataset from multiple sources such as from various hospitals to increase the dataset size and improve the machine learning model’s generalization ability.

In addition, we may also apply federated machine learning to keep identifiable medical data safe. Additionally, some techniques such as data augmentation [54], transfer learning [55], and few-shot learning [56] could be considered to address the issue of small dataset sizes. Thirdly, in terms of XAI evaluation, there is no real consensus. No objective and unified evaluation metric have been adopted. For general XAI evaluations, some researchers have proposed an evaluation approach. For example, Holzinger et al. presented a new approach on explaining quality, which was called the system causability scale (SCS) [53]. It utilized the Likert scale method and could quickly assess whether the explainable model was appropriate for its intended purpose. However, for medical XAI evaluation, we believe that this should be based on human-centered evaluation. More specifically, it should be evaluated by both medical experts and AI experts. For example, medical experts may evaluate the XAI method using the related clinical tasks to ensure the medical XAI application which can make the explainable clinical inference. In comparison, AI experts may evaluate the XAI applications on their generalization and robustness.

Finally, medical professionals should participate in the design and development stages of future studies of medical XAI applications. A good medical XAI application requires interdisciplinary collaboration. More specifically, medical experts should provide their prior medical knowledge, and their suggestions as well as feedback will help improve the design of AI algorithms. AI experts and data scientists should make sure that medical XAI applications can assist medical experts in making the explainable clinical inference. Consequently, we believe that the medical XAI models will become more acceptable to the medical domain. In order to achieve that, improving human–computer interactions (HCIs) is a promising approach. It will be feasible for both medical experts and AI experts to work together via a well-designed HCI medical application.

### 4.5. Research Questions

The research questions asked in the Introduction of this review are discussed here.

**RQ1:** What are the current research trends on medical XAI applications?
**Answer:** Based on the reviewed literature included in this review, we have found that most studies in the literature applied post hoc XAI methods. In general, they followed the pipeline which we have illustrated in Figure 3.
**RQ2:** How does the studies included in this review tackle the trade-off between accuracy and explainability?
**Answer:** We have summarized the surveyed studies and listed their AI evaluation metrics and XAI evaluations. In terms of AI performance, most of the studies performed well. However, only a few studies have provided XAI evaluations. In addition, most of the papers did not evaluate the model’s effectiveness by medical experts. Therefore, we cannot answer how these studies tackle the trade-off between accuracy and explainability.
**RQ3:** Is it possible to deploy these models in a clinical real-world environment to assist medical experts in making explainable clinical inferences?
**Answer:** Currently, there are still many limitations to medical XAI applications, and it is not feasible to deploy the models into the clinical environment. However, we believe that the future direction for medical XAI applications is promising.

## 5. Conclusions

In conclusion, this article has reviewed the existing literature and provided an in-depth survey of medical XAI applications in diagnosis and surgery. AI methods, XAI methods, XAI type, XAI evaluation and AI performance of the included papers in the survey are all discussed and compared. Additionally, we have presented an experimental showcase to illustrate how different XAI methods can be utilized in medical XAI applications. Moreover, we have provided a summary of the study and addressed the current limitations and future perspectives of medical XAI applications. In the interdisciplinary field of artificial intelligence and medicine, we believe that this review can reduce the gap between AI and medical professionals and provide helpful information for future researchers to design medical XAI applications.

## Figures and Tables

**Table 1:** Literature review of medical XAI applications in diagnosis.

SN#	Reference	Year	Aim	AI Algorithm	AI Evaluation Metrics	XAI Method	XAI Method Type	XAI Evaluation?
1	[24]	2021	Allergy diagnosis	kNN, SVM, C 5.0, MLP, AdaBag, RF	Accuracy: 86.39% Sensitivity: 75%	Condition-prediction (IF-THEN) rules	Rule-based	No
2	[25]	2021	Breast cancer therapies	Cluster analysis	N/A	Adaptive dimension reduction	Dimension reduction	No
3	[26]	2021	Spine	One-class SVM, binary RF	F1: 80 ± 12% MCC: 57 ± 23% BSS: 33 ± 28%	Local interpretable model-agnostic explanations (LIME)	Explanation by simplification	No
4	[27]	2021	Alzheimer’s disease	Two-layer model with RF	First layer: accuracy: 93.95% F1-score: 93.94% Second layer: 87.08% F1-score: 87.09%	SHAP, Fuzzy	Feature relevance, rule-based	No
5	[28]	2021	Hepatitis	LR, DT, kNN, SVM, RF	Accuracy: 91.9%	SHAP, LIME, partial dependence plots (PDP)	Feature relevance, explanation by simplification	No
6	[30]	2021	Chronic wound	CNN-based model: pretrained VGG-16	Precision: 95% Recall: 94% F1-score: 94%	LIME	Explanation by simplification	No
7	[31]	2021	Fenestral otosclerosis	CNN-based model: proposed otosclerosis-logical neural network (LNN) model	AUC: 99.5% Sensitivity: 96.4% Specificity: 98.9%	Visualization of learned deep representations	Visual explanation	No
8	[32]	2021	Lymphedema (Chinese EMR)	Counterfactual multi-granularity graph supporting facts extraction (CMGE) method	Precision: 99.04% Recall: 99.00% F1-score: 99.02%	Graph neural network, counterfactual reasoning	Restricted neural network architecture	No
9	[33]	2020	Clinical diagnosis	Entity-aware Convolutional neural networks (ECNNs)	Top-3 sensitivity: 88.8%	Bayesian network ensembles	Bayesian models	Yes
10	[34]	2020	Glioblastoma multiforme (GBM) diagnosis	VGG16	Accuracy: 97%	LIME	Explanation by simplification	No
11	[35]	2020	Pulmonary nodule diagnostic	CNN	Accuracy: 82.15%	Visually interpretable network (VINet), LRP, CAM, VBP	Visual explanation	No
12	[36]	2020	Alzheimer’s disease diagnosis	Naïve Bayes (NB), grammatical evolution	ROC: 0.913 Accuracy: 81.5% F1-score: 85.9% Brier: 0.178	Context-free grammar (CFG)	Rule-based	No
13	[37]	2020	Lung cancer diagnosis	Neural networks, RF	N/A	LIME, natural language explanation	Explanation by simplification, text explanation	No
14	[38]	2020	Traumatic brain injury (TBI) identification	k-means, spectral clustering, gaussian mixture	N/A	Quality assessment of the clustering features	Feature relevance	No
15	[39]	2020	COVID-19 chest X-ray diagnosis	CNN-based model: proposed COVID-Net	Accuracy: 93.3% Sensitivity: 91.0%	GSInquire	Restricted neural network architecture	No
16	[41]	2020	Colorectal cancer diagnosis	CNN	Accuracy: 91.08% Precision: 91.44% Recall: 91.04% F1-score: 91.26%	Explainable Cumulative Fuzzy Class Membership Criterion (X-CFCMC)	Visual explanation	Yes
17	[42]	2020	Diagnosis of thyroid nodules	Neural network	Accuracy: 93.15% Sensitivity: 92.29% Specificity: 93.62%	CAM	Visual explanation	No
18	[43]	2020	Phenotyping psychiatric disorders diagnosis	DNN	White matter accuracy: 90.22% Sensitivity: 89.21% Specificity: 91.23%	Explainable deep neural network (EDNN)	Visual explanation	No
19	[44]	2020	Parkinson’s disease (PD) diagnosis	CNN	Accuracy: 95.2% Sensitivity: 97.5% Specificity: 90.9%	LIME	Explanation by simplification	No
20	[45]	2019	Post-stroke hospital discharge disposition	LR, RF, RF with AdaBoost, MLP	Test accuracy: 71% Precision: 64% Recall: 26% F1-score: 59%	LR, LIME	Intrinsic, Explanation by simplification	No
21	[46]	2019	Breast cancer diagnostic decision and therapeutic decision	kNN, distance-weighted kNN (WkNN), rainbow boxes-inspired algorithm (RBIA)	Accuracy: 80.3%	Case-based reasoning (CBR) approach	Explanation by example	Yes
22	[47]	2019	Alzheimer’s diagnosis	RF, SVM, DT	Sensitivity: 84% Specificity: 67% AUC: 0.81	An interpretable ML model: sparse high-order interaction model with rejection option (SHIMR)	Rule-based	No

SN#: serial number; N/A: not applicable; AI: artificial intelligence; XAI: explainable artificial intelligence; kNN: k-nearest neighbor; SVM: support vector machine; MLP: multi-layer perceptron; RF: random forest; MCC: matthews correlation coefficient; BSS: brier skill score; SHAP: SHapley Additive exPlanations; LR: logistic regression; DT: decision tree; LIME: Local interpretable model-agnostic explanations; PDP: partial dependence plots; CNN: convolutional neural networks; DNN: deep neural network; AUC: area under the curve.

**Table 2:** Literature review of medical XAI applications in surgery.

SN#	Reference	Year	Aim	AI Algorithm	AI Evaluation Metrics	XAI Method	XAI Method Type	XAI Evaluation?
23	[48]	2020	Evidence-based recommendation surgery	XGBoost	Validation accuracy: 78.9%	SHAP	Feature relevance	No
24	[49]	2020	Surgery training	SVM	Accuracy: 92% Sensitivity: 100% Specificity: 82%	Virtual operative assistant	Feature relevance	No
25	[50]	2019	Surgical skill assessment	FCN	Suturing accuracy: 100% Needle passing accuracy: 100% Knot tying accuracy: 92.1%	CAM	Visual explanation	No
26	[51]	2019	Automatic recognition of instruments in laparoscopy videos	CNN	M2CAI Cholec data tuning on InstCnt non-instrument Instrument: Precision: 96% Sensitivity: 86% F1-score: 97%	Activation maps	Visual explanation	No
27	[52]	2019	Surgical education	CNN	Percentage of relevant frames among top 50 retrieved frames for three phases: 64.42%, 99.54%, 99.09%	Saliency map, content-based image retrieval	Visual explanation, explanation by example	No

SN#: serial number; AI: artificial intelligence; XAI: explainable artificial intelligence; SHAP: SHapley Additive exPlanations; SVM: support vector machine; FCN: fully convolutional neural network; CAM: class activation mapping; CNN: convolutional neural networks.
